# The VAR2CSA malaria protein efficiently retrieves circulating tumor cells in an EpCAM-independent manner

**DOI:** 10.1038/s41467-018-05793-2

**Published:** 2018-08-16

**Authors:** Mette Ø. Agerbæk, Sara R. Bang-Christensen, Ming-Hsin Yang, Thomas M. Clausen, Marina A. Pereira, Shreya Sharma, Sisse B. Ditlev, Morten A. Nielsen, Swati Choudhary, Tobias Gustavsson, Poul H. Sorensen, Tim Meyer, David Propper, Jonathan Shamash, Thor G. Theander, Alexandra Aicher, Mads Daugaard, Christopher Heeschen, Ali Salanti

**Affiliations:** 1 Centre for Medical Parasitology at Department of Immunology and Microbiology, University of Copenhagen and Department of Infectious Diseases, Copenhagen University Hospital, 2200 Copenhagen, Denmark; 2 Vancouver Prostate Centre, Vancouver, BC V6H 3Z6 Canada; 3 Department of Urologic Sciences, University of British Columbia, Vancouver, BC V5Z 1M9 Canada; 4 Stem Cells in Cancer & Ageing, Barts Cancer Institute, Queen Mary University of London, London, EC1M 6BQ United Kingdom; 5 Division of Urology, Department of Surgery, Tri-Service General Hospital, National Defense Medical Center, 11490 Taipei, Taiwan; 6 Department of Molecular Oncology, British Columbia Cancer Research Centre, Vancouver, BC V5Z 1L3 Canada; 7 UCL Cancer Institute, University College London, London, WC1E 6BT United Kingdom; 8 Department of Medical Oncology, Barts Health NHS, London, EC1A 7BE United Kingdom; 9 School of Medical Sciences, University of New South Wales, Sydney, NSW 2052 Australia

**Keywords:** Assay systems, Cancer screening, Tumour biomarkers

## Abstract

Isolation of metastatic circulating tumor cells (CTCs) from cancer patients is of high value for disease monitoring and molecular characterization. Despite the development of many new CTC isolation platforms in the last decade, their isolation and detection has remained a challenge due to the lack of specific and sensitive markers. In this feasibility study, we present a method for CTC isolation based on the specific binding of the malaria rVAR2 protein to oncofetal chondroitin sulfate (ofCS). We show that rVAR2 efficiently captures CTCs from hepatic, lung, pancreatic, and prostate carcinoma patients with minimal contamination of peripheral blood mononuclear cells. Expression of ofCS is present on epithelial and mesenchymal cancer cells and is equally preserved during epithelial–mesenchymal transition of cancer cells. In 25 stage I–IV prostate cancer patient samples, CTC enumeration significantly correlates with disease stage. Lastly, rVAR2 targets a larger and more diverse population of CTCs compared to anti-EpCAM strategies.

## Abstract (web summary)

Isolation of circulating tumor cells (CTCs) allows for non-invasive disease monitoring and characterization. Here the authors describe an alternative CTC isolation method based on the ability of the malaria rVAR2 protein to specifically bind oncofetal chondroitin sulfate, which is expressed by all cancer cells

## Introduction

Metastasis, the process in which malignant cells spread from the primary tumor to distant sites, is of key importance in cancer. Up to 90% of cancer-related deaths are related to the metastatic spread of cancer cells^1–3^. This complex process is vital to cancer progression and involves intravasation of cancer cells into the blood stream^2^. The cancer cells traveling in the blood are called circulating tumor cells (CTCs)^4,5^, and a subset of these has increased metastatic capacity^6^. CTCs have spurred increasing clinical interest since their levels in the blood were shown to be predictive of overall outcome for patients with metastatic colorectal, breast, prostate, and lung carcinomas^7–10^. Furthermore, the detection and enumeration of CTCs in patient blood samples, also termed liquid biopsies, provide a non-invasive tool for real-time monitoring of treatment response and estimating risk for metastatic relapse^11^. Besides enumeration, isolation of viable CTCs from blood enables individual and longitudinal molecular characterization and downstream experimental analysis, irrespective of the availability of tumor tissue biopsies. The ability to perform cellular analysis of bulk CTCs, but also contained subpopulations of cells with enhanced metastatic capacity, may represent a major advantage over DNA-based approaches, such as the detection of circulating tumor DNA^12^.

Several CTC detection and isolation platforms have been described^13^. Many recently developed systems are based on distinct biophysical properties of CTCs such as their theoretically larger size compared to peripheral blood mononuclear cells (PBMCs). However, studies have shown a large variation in CTC size and a considerable size overlap between CTCs and PBMCs^14^. Therefore, while these methods may provide viable CTCs, separation purely based on size could be too restrictive and introduce a considerable bias by missing important metastatic cells for the downstream analysis. Other systems for CTC isolation use antibodies to target epithelial markers, such as the epithelial cell adhesion molecule (EpCAM) cell surface protein. One of these is the CellSearch® CTC platform, which relies on detecting CTCs using anti-EpCAM antibody-coated magnetic ferrofluid nanoparticles followed by bulk magnetic enrichment^4^. In this platform, enriched cancer cells are identified as CTCs by their cytokeratin (CK) positivity using a fluorescent-labeled antibody, and potentially contaminating PBMCs are identified by a CD45 counterstain. This system represents the current gold standard for CTC enumeration and is approved by the US Food and Drug Administration (FDA) for monitoring patients with metastatic breast, colorectal, and prostate cancers^15^. Given the heterogeneous nature of CTCs, EpCAM-based capture approaches are inherently biased toward capturing CTCs with well-preserved epithelial traits and are rarely efficient in epithelial cancers with downregulated EpCAM expression, e.g., during epithelial–mesenchymal transition (EMT), or in cancers of mesenchymal origin (i.e., sarcomas)^16–18^. In an attempt to include these cells, many CTC isolation methods combine several antibodies in an antibody cocktail and thereby target a larger population of CTCs^19–21^. Such cocktails are, however, often only applicable to specific tumor types and prone to capture more non-cancer cells including white blood cells^22,23^. A similar contamination issue arises when the inverse approach is taken and CTCs are enriched by depletion of CD45-positive white blood cells, most likely due to a considerable fraction of leukocytes with low-level expression of surface marker^14,24,25^. Considering the limitations of the above-described methods, it is clear that the field would benefit greatly from a specific and universally expressed cancer marker for capturing and detecting CTCs.

The isolation of CTCs requires a highly specific target, which is completely absent from normal cells. In line with this, we have recently described a uniquely modified form of chondroitin sulfate (CS), termed oncofetal chondroitin sulfate (ofCS), which is expressed by placental cells and cancer cells of both epithelial and mesenchymal origin^26^. CS belongs to the family of glycosaminoglycans (GAG), which are long, linear carbohydrates made up of repeated disaccharide units that can be differentially modified by disaccharide sulfations. CS can be attached to different proteins called chondroitin sulfate proteoglycans (CSPGs) on the cell membrane or in the extracellular matrix. We have shown that ofCS can be attached to more than 30 different proteoglycans^26–30^. A single cancer cell may display different combinations of these ofCS proteoglycans and thereby ofCS serves as a more robust cancer biomarker as it is not restricted to the expression of a single protein. CSPGs are often overexpressed in primary as well as metastatic tumors, which, in combination with the expression of the specific ofCS structure across a diverse array of tumor types as well as its strict cancer specificity, makes them an appealing target for the universal and efficient isolation and detection of CTCs^31^.

We recently made the exciting discovery that the unique ofCS can be detected by the VAR2CSA malaria protein^26^. In placental malaria, parasite-infected erythrocytes adhere to ofCS in the placenta using the malaria VAR2CSA protein as an anchor^32^. Testing a wide range of cells and tissues, we found that the recombinant VAR2CSA (rVAR2) protein also binds more than 95% of cancer cell lines and tissues of epithelial, mesenchymal, and hematopoietic origin, with very limited binding to non-cancerous cells or normal tissue (besides placental tissue)^26^. This suggests that expression of ofCS is vital for the cellular attributes of embryonic and cancer cells, such as rapid proliferation, migration, and invasion^26^. We have shown that ofCS plays a key role in tumor cell motility through canonical integrin signaling pathways, and thus supports the metastatic potential of cancer cells^27^. In line with this, we found ofCS to be highly expressed in human metastatic lesions in situ and showed that rVAR2 could inhibit metastasis of cancer cells in mice^27^. As rVAR2 shows cancer-specific and origin-independent binding to ofCS both prior to and after the metastatic process, we hypothesized that rVAR2 could be a useful tool to broadly and efficiently capture rare cancer cells in complex blood samples.

Here, we present a CTC isolation method based on rVAR2 conjugated to 4.5 µm streptavidin-coated magnetic CELLection™ Biotin Binder Dynabeads®. Using the IsoFlux™ System to retrieve Dynabead-bound cells, we find a markedly enhanced CTC capture compared to EpCAM-based techniques in a diverse set of clinical blood samples. Importantly, our data confirm that the additionally captured subset of EpCAM-negative CTCs indeed derives from the respective tumor site. Our data indicate that the ofCS modification is independent of tumor type and cell differentiation status, demonstrating the potentially broad applicability of the rVAR2-based CTC isolation method.

## Results

### rVAR2 binds to epithelial and mesenchymal cancer cells

Based on the specific and high affinity binding of rVAR2 to ofCS on cancer cells^26^, we sought to establish a cancer-specific and tumor-type-independent CTC isolation method. To examine the potential use of rVAR2 for binding of cancer cells in blood samples, cancer cells of breast (MDA-MB-231, MCF7, Hs578T), prostate (LNCaP, PC3, DU145), colorectal (COLO205, HT-29, SW480), and lung carcinomas (A549) as well as osteosarcoma (U2OS) and melanoma (C32) were mixed with PBMCs in a 1:1 ratio. Flow cytometry analysis showed that rVAR2 bound specifically to cancer cells of epithelial and mesenchymal origin (Table 1). It should be noted that while rVAR2 binding to DU145 cells was stronger than binding to PBMCs, rVAR2 binding of the other metastasis-derived prostate cancer cell lines LnCAP and PC3 was even more pronounced.

**Table 1:** rVAR2 binding to cancer cells or peripheral blood mononuclear cells (PBMCs)

Tissue	Cell line	Cancer cells^a^ (Geometric MFI)	PBMCs^a^ (Geometric MFI)
Breast	MDA-MB-231	263	1.14
	MCF7	366	1.1
	Hs578T	440	1.4
Prostate	LNCaP	78.6	3.95
	PC3	320	0.77
	DU145	13.9	0.94
Colorectal	COLO295	409	2.75
	HT-29	207	6.15
	SW480	229	5.16
Others	A549	248	3.12
	U2OS	227	0.97
	C32	325	2.72
	PBMCs	No cells added	1.11

^a^Cancer cells were mixed with PBMCs in a 1:1 ratio, incubated with His-tagged rVAR2 in combination with anti-penta His Alexa Fluor 488 and analyzed by flow cytometry

**Fig. 1:**
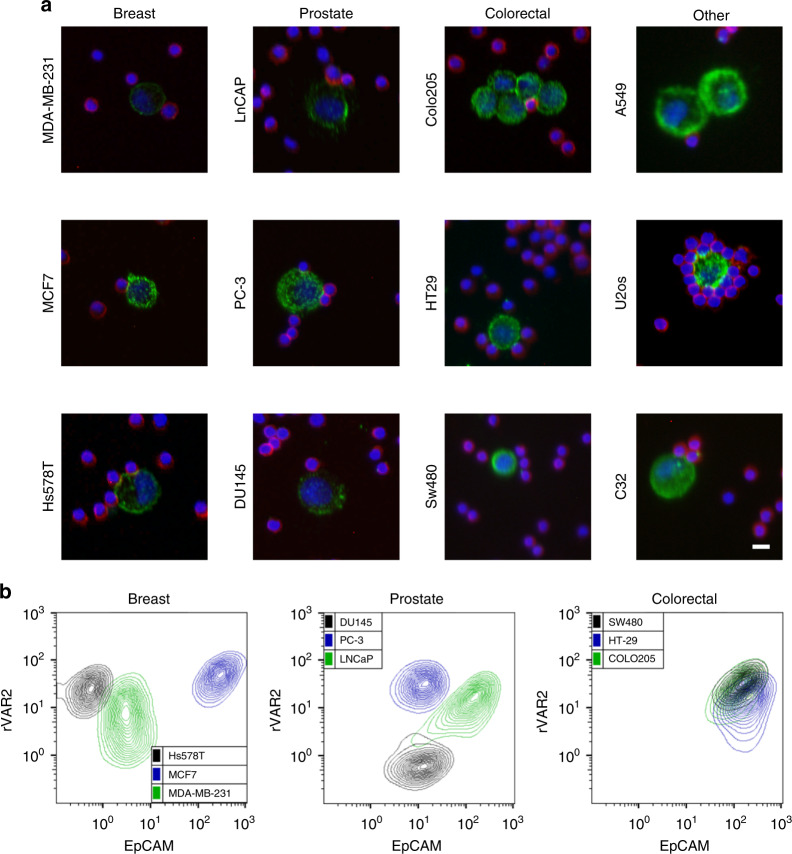
rVAR2 binds specifically to a diverse repertoire of cancer cells. **a** Detection of cancer cells using the CytoTrack platform. Representative confocal microscopy images of indicated cell lines. Cancer cells were mixed with PBMCs in a 1:5000 ratio prior to analysis and stained with His-tagged rVAR2 in combination with anti-penta His Alexa Fluor 488 (green), an anti-CD45 Cy5 antibody (red), and DAPI (blue). Scale bars, 10 µm. **b** Flow cytometry measured fluorescence intensity of three breast cancer (left panel), three prostate cancer (middle panel), and three colorectal cancer (right panel) cell lines stained by His-tagged rVAR2 in combination with anti-penta His Alexa Fluor 488 (*y*-axis) and a PE-conjugated anti-EpCAM antibody (*x*-axis)

Statistically, non-specific binding will dramatically increase when the number of non-target cells increases^33^. Therefore, we further verified the ability of rVAR2 to distinguish cancer cells from PBMC, by mixing cancer cells with PBMCs in a 1:5000 ratio and analyzing the samples for rVAR2 binding using a CytoTrack scanning device. The CytoTrack system allows for high throughput, multispectral confocal imaging of CTCs in blood samples^34^. The platform does not perform any prior enrichment of CTCs and therefore the enumeration of rare cells relies solely on specific biomarker detection using specific fluorescently labeled probes. Following staining with His-tagged rVAR2 in combination with an Alexa Fluor 488-conjugated anti-penta His antibody, the CytoTrack scanning device readily detected both epithelial and mesenchymal human cancer cells in a background of normal CD45-positive PBMCs (Fig. 1a).

These data indicate that an rVAR2-based CTC isolation method would be cancer specific and independent of the expression of epithelial markers. This was further confirmed by performing dual staining with rVAR2 and an anti-EpCAM antibody on nine different carcinoma cells lines, followed by flow cytometry analysis. As expected, rVAR2 binding did not correlate with the expression level of the epithelial marker EpCAM (Fig. 1b).

### rVAR2 binding is unaffected by phenotypic plasticity

**Fig. 2:**
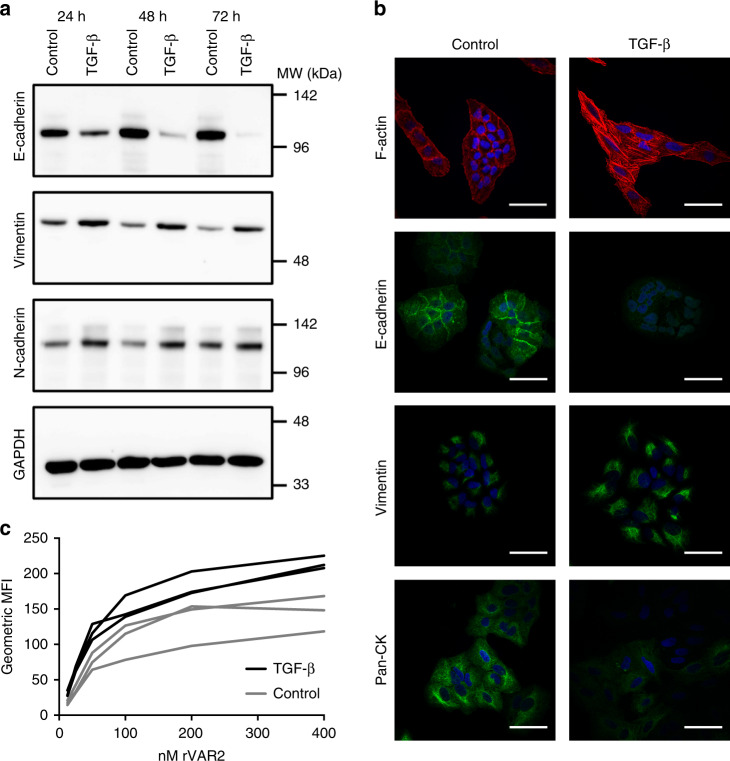
Epithelial–mesenchymal transition increases rVAR2 binding. **a** Western blot of A549 cell lysates after 24, 48, and 72 h of treatment with TGF-β or control (TGF-β dissolution buffer). Blots were incubated with rabbit anti-E-cadherin, anti-vimentin, anti-N-cadherin, or anti-GAPDH antibodies and detected by anti-rabbit HRP antibody. **b** Representative images of fixed A549 cells after 48 h of treatment with TGF-β or control buffer. Cells were incubated with DAPI, phalloidin alexafluor 594 to stain F-actin, anti-pan Cytokeratin Alexa Fluor 488, or rabbit anti-E-cadherin and anti-vimentin in combination with anti-rabbit FITC antibodies. Scale bars, 50 µm. **c** Intensity of rVAR2 staining (MFI) of A549 cells treated with TGF-β or control buffer for 48 h (*P* < 0.001, generalized least squares regression model). Binding of rVAR2 was detected by flow cytometry using anti-penta His Alexa Fluor 488 antibody. Three independent experiments were performed. MFI mean fluorescence intensity

Epithelial-mesenchymal transition (EMT) provides cancer cells with a phenotypic plasticity that is thought to be essential for the metastatic progression of primary carcinomas^35–38^. During this transition, epithelial surface markers, such as EpCAM, can be downregulated, rendering them less suitable targets for CTC isolation. To determine whether ofCS expression is maintained after the transition of a carcinoma cell toward a more mesenchymal phenotype, we induced EMT in the A549 lung adenocarcinoma cell line using TGF-β^39^. The induction of EMT following TGF-β treatment was confirmed by decreased expression of the epithelial marker E-cadherin, whereas expression of the mesenchymal markers vimentin and N-cadherin increased (Fig. 2a, b). Accordingly, the cells gained an elongated morphology and showed decreased expression of pan-CK, confirming their transition (Fig. 2b). Most importantly, rVAR2 binding was preserved, following induction of EMT as measured by flow cytometry (Fig. 2c). Preserved rVAR2 binding to cancer cells after EMT induction was also found for the human glioblastoma U87mg cell line (Supplementary Fig. 1). While EMT most likely drives the escape of cancer cells from the primary tumor site, the reverse process, termed mesenchymal–epithelial transition (MET), is thought to drive the colonization at the distant metastatic site^40,41^. To explore whether the mesenchymal–epithelial plasticity affects the expression of ofCS on cancer cells, we removed TGF-β from the culture media of EMT-induced A549 cells^42^. Seventy-two hours after the removal of TGF-β, mesenchymal markers, such as fibronectin and N-cadherin, were reduced, indicating that the A549 cells had returned to a more epithelial state (Supplementary Fig. 2a). Furthermore, the Snail EMT transcription factor, which was found upregulated as a consequence of TGF-β treatment, was reduced to background levels after the simplified MET (Supplementary Fig. 2a). Intriguingly, rVAR2 binding remained both through the EMT and MET (Supplementary Fig. 2b).

### rVAR2-coated beads capture cancer cells in blood samples

Having confirmed that rVAR2 can detect a range of different types of cancer cells in complex blood samples and that the binding to cancer cells is maintained during EMT/MET, we developed an rVAR2-based method to isolate CTCs. To allow easy isolation of viable CTCs, we used magnetic CELLection™ Biotin Binder Dynabeads® and biotinylated rVAR2 to isolate ofCS-positive CTCs on an IsoFlux™ microfluidic isolation system (Fluxion). Instruments combining microfluidics with magnetic micrometer-scale bead-based cell sorting have shown promising results in detecting rare cells in complex blood samples^14,43^. On the IsoFlux™ system, the cells are aligned within a microfluidic channel and directed across a magnetic field isolation zone at slow velocity^43^. This enables control of the time by which cells reside in the isolation zone, ensuring high recovery of magnetically labeled cells. In addition, unlabeled PBMCs are directed away from the isolation zone by gravitational and hydrodynamic forces, thereby improving the purity of the isolated CTC fraction^43^. The rVAR2 construct used in this study was based on our previously defined minimal ofCS binding region in the native full-length VAR2CSA protein, which stretches over 121 kDa^44^. This complicates direct conjugation of rVAR2 to beads, as it would likely interfere with the capacity of rVAR2 to interact with ofCS. To circumvent this problem, beads were coated with rVAR2 using a split protein conjugation system (SpyTag/SpyCatcher) in a four step process^45,46^. First, rVAR2 was genetically modified to include an N-terminal 13 amino acid SpyTag peptide. Second, the corresponding 13 kDa SpyCatcher protein was produced recombinantly and biotinylated. Third, SpyTagged rVAR2 and the biotinylated SpyCatcher were mixed resulting in the formation of an isopeptide bond between the two proteins. Finally, this biotinylated complex was immobilized on streptavidin-covered magnetic Dynabeads®. This procedure allowed conjugation of rVAR2 to magnetic beads without abolishing the ofCS-binding capacity of rVAR2.

**Fig. 3:**
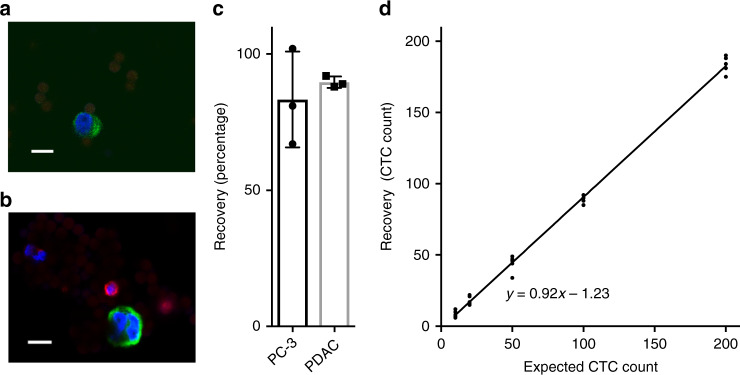
rVAR2-based CTC isolation using magnetic beads. **a** Representative confocal microscopy image of a PC3 cell captured by rVAR2-coated magnetic beads in combination with the IsoFlux™ device after spiking into 5 mL of healthy donor blood. Isolated cells were incubated with anti-cytokeratin FITC antibody (green), anti-CD45 PE antibody (red), and DAPI (blue). Scale bars, 10 µm. **b** A low-passage pancreatic ductal adenocarcinoma (PDAC) cell from a patient-derived xenograft captured by rVAR2-coated magnetic beads in combination with the IsoFlux™ device after spiking into 5 mL of healthy donor blood. Isolated cells were incubated with anti-cytokeratin FITC antibody (green), anti-CD45 PE antibody (red), and DAPI (blue). Scale bars, 10 µm. **c** CTC isolation efficiency by spike-in experiments; 100 PC3 cells or PDAC cells were spiked into 5 mL of blood and isolated by rVAR2-coated beads in combination with the IsoFlux™. The recovery was estimated by immunofluorescence staining of CK+, CD45−, DAPI+ cells (mean ± s. d., *n* = 3). **d** Recovery of PDAC cells spiked into 5 mL of blood. The number of spiked PDAC cells (10, 20, 50, 100, or 200 cells) is plotted versus the number of PDAC cells isolated by rVAR2-coated beads (*n* = 5). Equation shows a linear regression model

To test the ability of the rVAR2-coated magnetic beads to capture cancer cells, we spiked 100 PC3 prostate cancer cells into 5 mL of healthy donor blood and assessed cancer cell retrieval. First, red blood cells were eliminated by lysis, followed by a brief incubation of the rVAR2-coated beads with the cell sample. The cell-bead suspension was loaded onto the IsoFlux™ microfluidic cartridge and automatically processed in the IsoFlux™ instrument. Captured cells were manually transferred to slides, immobilized by a strong magnet, and stained for pan-CK, CD45, and 4′, 6-diamidino-2-phenylindole (DAPI). Cancer cells, defined as CK+, CD45−, DAPI+, were enumerated using the Ariol System software. Isolated PC3 prostate cancer cells showed clear CK staining (green) and no CD45 staining (red) (Fig. 3a). Since spike-in experiments with established cell lines rather poorly represent the heterogeneity of CTCs, low-passage pancreatic ductal adenocarcinoma (PDAC) cells from patient-derived xenografts (PDX) were used for further validation of the method. The isolated PDAC cells were easily distinguished from white blood cells by their CK+, CD45−, DAPI+ profile (Fig. 3b). To evaluate the isolation method, we performed an exact enumeration of three separate spike-in experiments using 100 cells. On average, the isolation recovery was 83% and 90% for the PC3 and PDAC cells, respectively (Fig. 3c). To further validate the efficiency of the rVAR2-based CTC recovery, we assessed the recovery after adding 10, 20, 50, 100, 200, or 500 PDAC cells to 5 mL blood. The average percentage of recovered cells was between 86.0 and 91.9% and the recovery did not vary systematically with the number of cells added (correlation coefficient (*R*^2^) = 0.996) (Fig. 3d and Table 2). Finally, we tested the sensitivity of cancer cell recovery in a more challenging setup by spiking 5 mL of blood with only three or six GFP-expressing PDAC cells. The average recovery for five replicates was 60.0% and 76.7% for three and six cells spiked in, respectively (Table 2). Collectively, these results show that rVAR2-coated beads enable efficient isolation of cancer cells spiked into a complex blood sample, demonstrating the high sensitivity of the procedure.

**Table 2:** Accuracy of rVAR2-based recovery of PDAC cells spiked into 5 mL of blood from healthy donors

PDAC cells added	Average recovery (%)	S.E.M. (*n*=5)
3^a^	60.0	12.5
6^a^	76.7	8.5
10	86.0	10.8
20	91.0	7.0
50	88.0	5.3
100	88.6	1.2
200	91.9	1.3
500	88.8	4.9

^a^The accuracy of recovery of three and six cells were done using GFP-expressing PDAC cells spiked into 5 mL of blood from healthy donors (*n* = 5). The number of cells used for spiking was visually confirmed before and after aspiration. Spiked samples were enriched for cancer cells using rVAR2-coated beads in combination with the IsoFlux™ and enumerated based on the detection of the GFP signal.

S.E.M. standard error of the mean

### rVAR2-coated beads capture CTCs in patient blood samples

**Fig. 4:**
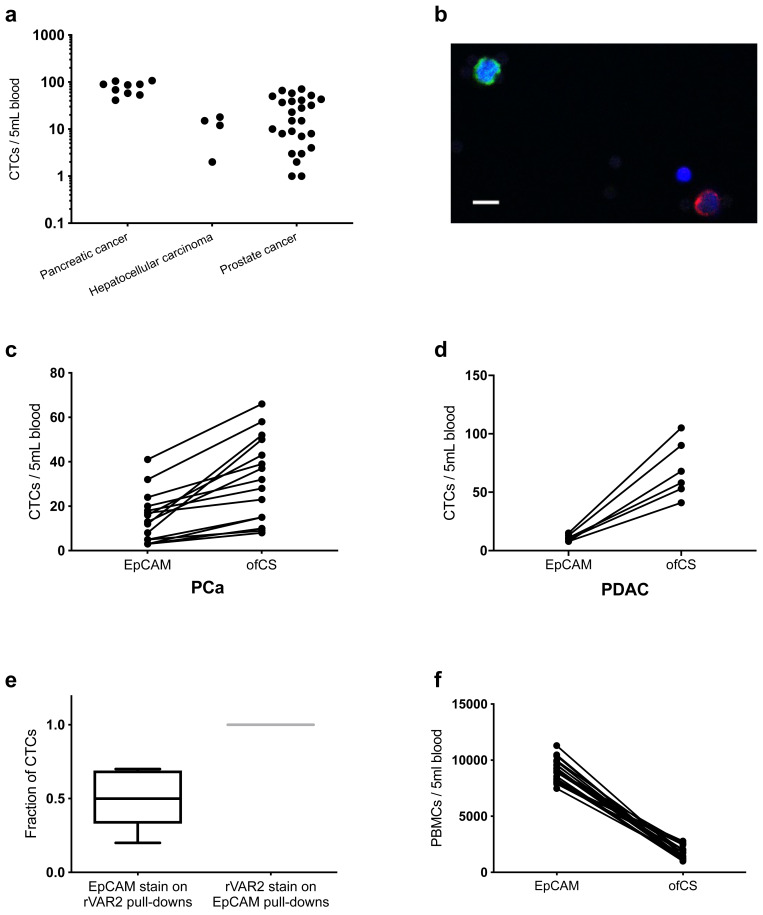
rVAR2- and EpCAM-based CTC isolation and enumeration in cancer patients. **a** Number of CTCs isolated from 5mL pancreatic (*n* = 9), hepatocellular (*n* = 4), and prostate (*n* = 25) cancer patient-derived blood using rVAR2-coated beads. CTCs were enumerated by immunofluorescence stainings and defined as CK+ CD45− DAPI+. **b** Representative confocal microscopy image of a circulating tumor cell isolated with rVAR2 from blood derived from one of the pancreatic cancer patients (patient 4, Table 3). Isolated cells were stained with anti-cytokeratin FITC antibody (green), anti-CD45 PE antibody (red), and DAPI (blue). Scale bar, 10 μm. **c** Number of CK+ CD45− DAPI+ CTCs isolated using rVAR2 or anti-EpCAM antibody-coated beads from 5mL blood from 15 of the stage II–III prostate cancer (PCa) patients (P < 0.02, Wilcoxon test for paired data). **d** Number of CK+ CD45− DAPI+ CTCs isolated using rVAR2 or anti-EpCAM antibody-coated beads from 5mL blood from six of the stage III–IV pancreatic ductal adenocarcinoma (PDAC) patients. **e** Box-Whiskers plot showing post-isolation characterization of CK+ CD45− DAPI+ CTCs using EpCAM or rVAR2 stain on CTCs isolated using rVAR2 (*n* = 7) or anti-EpCAM antibody-coated (*n* = 7) beads, respectively. The median is presented as the center line, whiskers as min to max values, and the 25th to 75th percentiles define the box. **f** Number of PBMCs contaminating the isolated CTCs from patient-matched blood samples using rVAR2 or anti-EpCAM antibody-coated beads. PBMC levels were estimated by immunofluorescence stainings and defined as CK−, CD45+, DAPI+ stained cells (P < 0.0001, Wilcoxon test for paired data) (*n* = 23).

In order to test whether rVAR2-coated beads enabled the isolation of CTCs from clinical samples, we analyzed blood samples from patients with pancreatic (*n* = 9), hepatic (*n* = 4), prostate (*n* = 25), and lung (*n* = 6) cancer at different stages of disease. rVAR2-coated beads captured CK+, CD45−, DAPI+ cells in all four cancer types (Fig. 4a–c), whereas no CTCs were detected in blood samples from healthy donors (*n* = 16).

Next, we sought to confirm that the isolated CK+, CD45−, DAPI+ cells originated from the diagnosed tumor of the given patient and thus were genuine CTCs. This was demonstrated in four of the samples from patients with PDAC. As four of these patients harbored activating G12 *KRAS* mutations, the copies of mutated DNA in the total CTC isolate could be quantified using highly sensitive digital droplet polymerase chain reaction (ddPCR). ddPCR was performed on genomic DNA material directly retrieved from the enumeration slide by microdissection. As a positive control, we used the DNA extract from 100 primary PDAC cells carrying the same mutation plus 5000 PBMCs. Table 3 summarizes the enumeration data as well as the ddPCR mutation analysis. The G12 *KRAS* mutation numbers for each of the four tested samples correlated with the CTC enumeration data, indicating that the captured CTCs, defined by a CK+, CD45−, DAPI+ profile, did indeed carry the *KRAS* mutation and thus originated from the pancreatic tumor (Table 3).

**Table 3:** *KRAS* mutational analysis of total DNA extract from CTC isolates from pancreatic cancer patient blood samples^a^

ID^a^	CTCs^b^	PBMCs^c^	*KRAS* mutation	Mutated *KRAS* genes (copies per µL)^d^
Patient 1	68	1170	G12D	0.94
Patient 2	87	1885	G12D	1.03
Patient 3	90	1075	G12V	1.05
Patient 4	107	1072	G12D	1.13

^a^Blood samples derive from the same pancreatic cancer patients as in Fig. 4a

^b^CTCs were defined as CK+, DAPI+, CD45−

^c^PBMCs were defined as CK−, DAPI+, CD45+

^d^For the analysis of each patient sample, 100 PDAC cells were spiked into 5000 PBMCs prior to the DNA extraction procedure and run in parallel as a positive control, resulting in values between 0.93 and 0.94 copies of mutated *KRAS* genes per microliter

### rVAR2 captures more CTCs and less PBMCs

Our data suggest that rVAR2 bears the potential for capturing a subset of non-epithelial CTCs in addition to the classical epithelial CTCs. To directly compare our ofCS-targeting CTC isolation method with the more common EpCAM-targeting strategy, we aimed to capture CTCs in a subset of the blood samples from lung, prostate, and pancreatic cancer patients using either rVAR2-coated or anti-EpCAM antibody-coated beads on the IsoFlux™ system. The rVAR2-based method detected higher CTC numbers than the EpCAM-based method in all patient-matched blood samples (Fig. 4d–f). On average the rVAR2-based CTC isolation resulted in a 5.3×, 2.8×, or 6.4× higher CTC levels for lung (*n* = 4), prostate (*n* = 15), and pancreatic (*n* = 6) cancer, respectively.

To characterize the isolated prostate CTCs, we stained rVAR2-captured CTCs for EpCAM and anti-EpCAM antibody captured CTCs for ofCS (using rVAR2). Only half of the rVAR2-captured CTCs were EpCAM positive, whereas all cells retrieved by the EpCAM-based method were ofCS positive as determined by rVAR2 staining (Fig. 4g). As expected, these results support the notion that the rVAR2-based CTC isolation results in the capture of a broader spectrum of CTCs and that none of the EpCAM-based captured CTCs are missed by rVAR2.

In order to confirm that the additional CK-positive cells isolated by rVAR2 were indeed CTCs, we compared the concentration (copies per microliter) of mutated *KRAS* genes in four of the PDAC isolates by performing ddPCR on the material from the CTC enumeration slide as described above. The mutational analysis was consistent with the CTC enumeration, showing an average of seven times higher levels of G12 *KRAS* mutation in the rVAR2 isolates compared to the EpCAM isolates (Table 4). Likewise, a higher average mutant allele fraction (MAF) for *KRAS* was found for the rVAR2-based CTC isolations (7.5%) than the EpCAM-based CTC isolations (1.1%). The higher MAF of the rVAR2-based CTC isolations most likely reflects the higher number of isolated CTCs as well as a lower PBMC contamination (Fig. 4h). On average, the number of contaminating PBMCs was 82% less after capturing ofCS-positive CTCs compared to the EpCAM-based capture strategy (*P* < 0.0001, Wilcoxon test for paired data) (*n* = 23).

**Table 4:** *KRAS* mutational analysis of total DNA extract from rVAR2- versus EpCAM-based CTC isolates

ID	*KRAS* mutation	rVAR2 isolation		EpCAM isolation	
		**CTCs**	***KRAS*** **mut. copies per μL**	CTCs	*KRAS* mut. copies per μL
Patient 1	G12D	53	0.65	10	0.10
Patient 2^a^	G12D	68	0.76	8	0.09
Patient 3	G12V	90	0.85	13	0.14
Patient 4	G12D	105	0.98	15	0.12

^a^Same patient as patient 1 in Table 3 but normalized to respective analysis

To expand on these findings, we confirmed the presence of mutated *KRAS* at a single cell level. rVAR2-isolated cells from two of the pancreatic cancer blood samples were stained for EpCAM in addition to the routine CK and CD45 used for detection. To ensure high cell-picking efficiency, we applied the single cell isolation workflow developed by Neumann et al.^47^. The bead-coated cells were placed on a glass slide and processed using the automated micromanipulator CellCelector (ALS, Jena, Germany). A magnetic field kept the cells in situ and five EpCAM-positive (EpCAM+, CK+, CD45−, DAPI+) and five EpCAM-negative (EpCAM−, CK+, CD45−, DAPI+) single cells were selected based on morphological criteria. Preference was given to individual cells with small round shape and an individual nucleus without signs of DNA fragmentations. Total RNA from picked single cells was isolated and used for cDNA synthesis. Following preamplification, the presence of *KRAS* mutations was verified by ddPCR. All EpCAM-positive and EpCAM-negative cells carried the expected *KRAS* mutation (Supplementary Fig. 3).

As our results indicated that rVAR2-captured CTCs could contain a mesenchymal subpopulation, we stained CTC enriched samples from two lung cancer patients for the mesenchymal marker vimentin. Samples from both patients contained cells that were double positive for CK and vimentin, strongly supporting our hypothesis that rVAR2 efficiently captures CTCs with an intermediate epithelial and mesenchymal phenotype (Fig. 4i). Notably, a minor fraction of the double-negative cells (CK−, CD45−, DAPI+) was found to be vimentin positive, suggesting the presence of mesenchymal CK− CTCs within the rVAR2-enriched cell population.

### rVAR2 can be used for CTC isolation in early-stage cancer

**Fig. 5:**
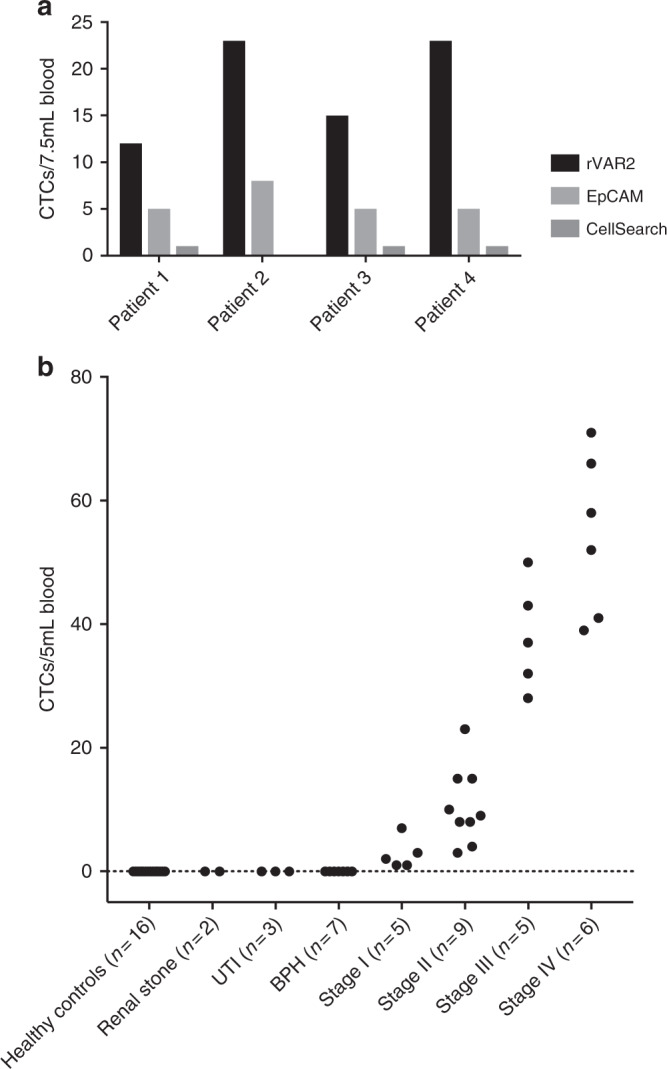
rVAR2-capture of CTCs from prostate cancer patients. **a** Number of CK+ CD45− DAPI+ CTCs isolated from 7.5 mL blood from four prostate cancer patients using rVAR2 or anti-EpCAM antibody-coated beads or the CellSearch® CTC platform. **b** CTC enumeration using rVAR2-coated beads on blood samples from prostate cancer patients with different disease stages (*n* = 25) as well as from healthy controls (*n* = 16) and patients with non-malignant diseases (*n* = 12). (*P* = 0.0001 for association between disease severity and CTC number, Kruskal–Wallis test). UTI: urinary tract infection, BPH: benign prostatic hyperplasia

To test our rVAR2-based capture of prostate CTCs against the current clinical practice, four patient-matched blood samples were also analyzed on the CellSearch® CTC platform. The EpCAM capture on this platform did not detect CTCs above the pre-specified cut off (≥2 CTCs per 7.5 mL blood) in any of the tested samples (Fig. 5a). However, it should be noted that the CellSearch® CTC platform was validated in metastatic prostate cancer while we in this case tested stage II prostate cancer patients^9^. Nevertheless, the EpCAM-based capture analyzed on the IsoFlux™ detected 5, 8, 5, and 5 CTCs in the patient-matched samples (estimated from the 5 mL capture as shown in Fig. 4d). Interestingly, the rVAR2-based detection method captured the highest number of cells with 12, 23, 15, and 23 CTCs, respectively, in the four patient samples (estimated from the 5 mL capture as shown in Fig. 4d). Thus, considerably more CTCs were isolated using rVAR2.

To test whether the rVAR2 method had the potential to predict disease stage, rVAR2-based CTC numbers from prostate cancer patient blood samples (*n* = 25 from Fig. 4a) were classified according to the disease stage of the patient. CTCs were isolated from all patients, including all those in stage I and II (*n* = 14), and the number of captured CTCs showed a statistically significant association with patient disease stage (*P* = 0.0001, Kruskal–Wallis rank test) (Fig. 5b). Importantly, no CTCs were detected in blood from any of the healthy controls (*n* = 16) or patients with different non-malignant diseases (*n* = 12). Intriguingly, these data suggest that the rVAR2-based method could be used for CTC detection and staging in low-grade disease stages.

## Discussion

We have previously shown that ofCS is presented by nearly all cancer cells^26^. This suggests that ofCS could be an ideal target for the isolation of CTCs in a wide range of cancer types. In this study, we show that the rVAR2 malaria protein specifically detects ofCS on a variety of cancer cells in complex blood samples. Based on this, we developed a highly efficient CTC isolation protocol using the ofCS-targeting rVAR2 protein and magnetic beads in combination with the IsoFlux™ system. The analytical performance was evaluated and showed excellent recovery when cell lines and more heterogeneous primary cancer cells, ranging in numbers from 3 to 500 cells, were spiked into 5 mL blood from healthy donors. Importantly, this protocol enabled isolation of CTCs from pancreatic, hepatic, lung, and prostate cancer patients at various stages of disease, illustrating the broad applicability of the rVAR2-based CTC isolation method. Furthermore, we showed that rVAR2 resulted in a higher CTC yield in blood samples from prostate, pancreatic, and lung cancer patients as compared to EpCAM-based isolation of patient-matched samples.

It is interesting that rVAR2 captures more CTCs in all tested blood samples. One explanation could be the wide distribution of ofCS on cancer cells. ofCS is a secondary modification to a wide range of different CSPGs, of which multiple are co-expressed on different cancer cells. rVAR2 isolation of CTCs is therefore not dependent on the expression of a single marker^27^. It is likely that the expression of ofCS on multiple membrane bound proteins increases the density of target antigen on the cell surface and thereby improves the sensitivity of the rVAR2-based CTC isolation strategy compared to that of targeting a single protein, like EpCAM, which is more sensitive to protein up or downregulation. Another explanation for the increase in CTC recovery is that ofCS is simply present on a broader spectrum of the patient CTCs. This is substantiated by the fact that only a fraction of the rVAR2-captured CTCs showed EpCAM positivity, while all the EpCAM-captured CTCs were ofCS positive. The EpCAM−, CK+, CD45− cells captured by rVAR2 were confirmed to be genuine CTCs by *KRAS* mutation analysis on single cells from pancreatic cancer patient blood samples. While most carcinomas are considered EpCAM positive, the expression of EpCAM is often heterogenous within the tumor. The intratumoral heterogeneity as well as a potential transition toward a more mesenchymal phenotype could partly explain why EpCAM-based methods only detect a fraction of the CTCs, despite the epithelial origin of the tumor. In contrast, we show that rVAR2 binding to cancer cells is maintained after induction of EMT. In line with this, we demonstrate that the rVAR2-enriched CTC population from cancer patients contains vimentin-positive cells as well as EpCAM-negative cells, indicating that the rVAR2 isolates include mesenchymal-like subpopulations. Interestingly, the observed ofCS display on mesenchymal-like carcinoma cells is in accordance with our recently published work where we demonstrated that ofCS plays a key role in cancer cell motility through integrin signaling pathways, and thus seems to be a requirement for cancer cells to invade and metastasize^27^. A complete analysis of the mesenchymal subsets of CTCs is, however, beyond the scope of this study. As CK represents a validated marker for CTC detection, we chose to stain the bead-captured cells and define CTCs based on being CK+, DAPI+, and CD45−. Therefore, the CTC enumeration used for this study is still dependent on the expression of the epithelial marker, CK. As shown for the A549 lung cancer cell line, CK is also frequently downregulated during EMT and this has also been noted for the contained subset of cancer stem cells^6^. Thus, it is likely that capturing CTCs with rVAR2 followed by CK detection will miss certain subsets of CTCs. Intriguingly, Vim+, CK−, CD45− cells were detected in two blood samples from non-small cell lung cancer (NSCLC) patients and they may represent mesenchymal CTCs. Future studies will have to define and further validate the nature of the captured vimentin+, CK− putative CTCs. Collectively, our data suggest that rVAR2 specifically binds cancer cells regardless of their state of epithelial differentiation, and thus will enable the isolation of additional subsets of CTCs. These results do, however, not rule out that rVAR2− EpCAM+ CTC may exist, as suggested by the weak rVAR2 staining on DU145 cells (Fig. 1b). Future studies could study the combined use of EpCAM and rVAR2 for capturing CTCs to examine the potential cumulative effect.

In addition to giving higher CTC yields, the rVAR2-based isolation also resulted in a low contamination of PBMCs compared to the EpCAM-based isolation on the IsoFlux™ system. The number of CD45+, CK−, DAPI+ PBMCs in the rVAR2 isolates was not affected by disease stage, and healthy as well as non-malignant disease subjects showed equal numbers of PBMCs. Despite the overexpression of CSPGs on cancer cells, CS in general is present on all cells including PBMCs. However, the decrease in PBMC contamination supports our previous findings that rVAR2 exhibits a high specificity toward the cancer-specific ofCS modification^26^. In combination with the high CTC levels, the relative purity of the CTC isolates makes the rVAR2-based method suitable for downstream analysis such as whole-genome sequencing.

Currently, the only FDA-approved CTC detection platform, CellSearch®, is based on positive EpCAM selection of cells in patient blood. Our test of blood samples from four prostate cancer patients on the FDA-approved CellSearch® CTC platform yielded only one or no CTCs per sample. This is below the threshold for abnormality set forth for the instrument^4^. However, the CellSearch® CTC platform was validated in metastatic prostate cancer while we in this case tested stage II prostate cancer patients. Surprisingly, significantly more CTCs were isolated using the same isolation target, EpCAM, on the IsoFlux™ system. Direct comparison of the CTC isolation by the two different platforms is, however, difficult due to the use of different antibody clones and magnetic beads for capture. The IsoFlux™ system utilizes micrometer-scale beads which have shown to result in a magnetic moment that is sufficient for capturing cells even with low target expression^43^. This is likely to increase the sensitivity compared to the nanoscaled magnetic particles used in the FDA-approved CellSearch® CTC platform^43^. Also, the IsoFlux™ system uses microfluidic flow based enrichment of magnetically labeled cells, which increases the purity of the final output compared to no-flow bulk isolation^43^. Furthermore, differences in the validation stainings and the criteria used when assigning an object as a CTC could potentially affect the analytical interpretation of the result^33^. Thus, there are important technical differences between the two instruments that may explain at least in part the differences in CTC counts.

The data from this study clearly indicate that rVAR2 combined with the IsoFlux™ system results in markedly higher assay sensitivity, which could be an important factor in the clinic, as it reduces the number of cancer cases with false negative tests and potentially allows for CTC detection at earlier stages of disease. In addition, the increased sensitivity could make the rVAR2-based method suitable for risk monitoring in patients suffering from cancer types with reportedly low CTC levels as well as detection of minimal residual disease after therapy or surgical resection of a tumor. To further explore the clinical potential of the rVAR2-based method, CTC enumerations in 25 prostate cancer patients were associated with disease stage. Despite the relatively low number of patient samples available, the difference in levels of CTCs between the four stages of disease was remarkable. Thus, rVAR2 could potentially provide means for assessing the stage of the disease. However, large-scale prospective clinical trials are warranted to validate the clinical sensitivity as well as prognostic value of this CTC capture method.

In conclusion, we describe an efficient and cancer-specific method for the isolation of CTCs in complex blood samples using a recombinant VAR2CSA malaria protein. We show that rVAR2 specifically detects ofCS, a uniquely modified form of CS, on a wide variety of cancer cells, regardless of tumor origin. The rVAR2-based CTC isolation method showed markedly increased CTC retrieval compared to EpCAM-based techniques when testing blood from prostate, lung, and pancreatic cancer patients. Taken together, the rVAR2-based method not only provides a more sensitive and universal tool for CTC detection with the potential of a high clinical impact, but also allows for isolation of more, if not all, subsets of CTCs, which is of great value for downstream cellular analysis. This will hopefully provide further insights into the cellular characteristics of the highly metastatic subpopulation of cancer cells circulating in the blood of cancer patients, and thereby improve our understanding of metastasis formation and potentially enable the development of more efficient therapies.

## Methods

### Cell cultures

Sw480, COLO205, LNCaP, C32, Hs578t, and PDX-derived PDAC cells were cultured in RPMI 1640, whereas A549, PC-3, DU145, U2OS, and MDA-MB-231 were cultured in DMEM, HT-29 in McCoy’s, and MCF7 in EMEM with an additional supplement of 0.01 mg/mL insulin. All media for the cancer cell cultures were purchased from Sigma Aldrich and supplemented with 10% FBS, L-glutamine, penicillin, and streptomycin. All commercial cell lines originated from ATCC®. We regularly tested for mycoplasma contamination and none of the cell lines used in this paper have tested positive. Due to limitations of the in-house facilities, authentication of each cell line by genotyping is still in progress. None of the cell lines used in this paper are listed in the database of commonly misidentified cell lines maintained by ICLAC. For retrieval of the PDX-derived PDAC cells, human tissue was obtained with written informed consent from all patients and expanded in vivo as PDX. PDX-354 was processed as previously described^48^. Briefly, PDX-derived tumors were minced and enzymatically digested with collagenase (STEMCELL Technologies) for 90 min at 37 °C, and after centrifugation for 5 min at 1200 rpm, cell pellets were resuspended and cultured in RPMI (Invitrogen) supplemented with 10% FBS and 50 U/mL penicillin–streptomycin. Primary cultures were tested for mycoplasma contamination every 2 weeks (MycoAlert™ Mycoplasma Detection Kit, Lonza).

### Production of rVAR2

The subunit DBL1-ID2a of VAR2CSA (rVAR2) was recombinantly expressed in *Escherichia coli* as previously described^49^. In brief, the FCR3 DBL1-ID2a with a C-terminal V5 tag, penta-His tag, and a split protein tag sequence was inserted into a modified pET15b plasmid (Novagen) and transformed to SHuffle T7 Express Competent *E. coli* cells (New England Biolabs, C3029H). Following lysis of the cell pellet, rVAR2 expressed in a soluble form was purified by immobilized affinity chromatography (IMAC) followed by size-exclusion chromatography. Purity of the protein was confirmed by SDS page and Western blot, whereas specificity toward ofCS was ensured in ELISA and on cancer cells using flow cytometry.

### Flow cytometry

Blood samples were collected in CPDA Vacuette tubes and PBMCs were isolated using a Lymphoprep gradient. PBMCs were mixed with cancer cells in a 1:1 ratio and incubated with 250 nM rVAR2 or as otherwise indicated for 30 min at 4 °C. Following three wash steps in PBS with 2% FBS, cells were incubated with anti-penta His Alexa Fluor 488 (Cat. No. 35310, Qiagen, 1:500)^50^ and data were acquired using a FC500 flow cytometer (Beckmann Coulter). Secondary antibody controls were included in all experiments by adding only anti-penta His Alexa Fluor 488 without the His-tagged rVAR2. EpCAM levels in cancer cell lines were detected by an anti-human EpCAM antibody [VU-1D9] PE (Cat. No. ab112068, Abcam, 1:75)^51^. Mean fluorescence intensities (MFIs) were analyzed using FlowJo™ software.

### Induction of EMT

A549 cells were seeded at a density of 3000 cells/cm^2^ in DMEM supplemented with 10% FBS, L-glutamine, penicillin, and streptomycin. After attachment, cells were starved in 0.5% FBS for 24 h. Cells were subsequently treated with 10 ng/mL TGF-β (Cat. No. phg9214, Life Technologies) or TGF-β suspension buffer as control (40 mM acetic acid, 0.1% BSA in ultra-pure water) for 24, 48, or 72 h to induce EMT. Transition was confirmed by morphology changes and a change in expression of epithelial and mesenchymal markers using Western blot and immunofluorescence studies. For the MET studies, TGF-β was replaced by DMEM with 0.5% FBS after 72 h EMT induction and the partial return of the A549 cells to their epithelial state was observed for another 72 h.

For Western blots, cells were lysed with EBC lysis buffer for 30 min and the protein extract was balanced using a Bradford assay. Equal amount of protein lysates were loaded onto a NuPAGE Bis-Tris gel (ThermoFisher Scientific) after which samples were transferred to a nitrocellulose membrane (Biorad). Transfer was confirmed using Ponceau red staining and the membranes were blocked in 5% skimmed milk powder in TBS-T. Anti-GAPDH (14C10) antibody (Cat. No. 2118, Cell Signaling, 1:1000)^52^, anti-fibronectin antibody (Cat. No. 610077, BD Biosciences, 1:2000)^53^ or anti-E-cadherin (1:1000), anti-vimentin (1:1000), anti N-cadherin (1:500), anti-Snail (1:500), and anti-β-catenin (1:500) primary antibodies from the EMT Antibody Sampler Kit (Cat. No. 9782S, Cell Signaling)^54^ were added to the membrane in TBS-T supplemented with 2% skimmed milk powder and incubated overnight at 4 °C. To reduce antibody levels, the blots were cut into smaller lanes based on the molecular target size prior to antibody incubation (Supplementary Fig. 4). Following three washes in TBS-T, the membranes were incubated with anti-rabbit HRP (Cat. No. 9782S, Cell Signaling, 1:1000)^54^ for 1 h at room temperature and the reactivity was detected using LumiGlo Reserve Chemiluminescent Substrate (KPL).

For immunofluorescence studies, cells were grown on glass slides and fixed in 4% formaldehyde, washed three times in PBS, blocked with 1% BSA, 5% FBS, and 0.3% Tween in PBS for 1 h at room temperature, incubated overnight at 4 °C with anti-pan CK Alexa Fluor 488 (Ca. No. 53-9003-82, eBioscience, 1:500) or primary anti-E-cadherin and anti-vimentin antibodies from the EMT Antibody Sampler Kit (Cat. No. 9782S, Cell Signaling, 1:200)^55^ made in PBS with 1% BSA and 0.3% Triton X-100. After three washes in PBS, the fixed cells were incubated with secondary Fluorescein (FITC) Anti-Rabbit IgG Antibody (Cat. No. FI-1000, Vector Laboratories, 1:200)^56^ for 1 h at room temperature. For analysis of F-actin, cells were blocked in 1% BSA and stained with Alexa Fluor® 594 Phalloidin (Cat. No. A12381, ThermoFisher, 1:40)^57^ for 20 min at room temperature. All cells were stained with DAPI (Cat. No. D1306, Life Technologies)^26^ and mounted using FluorSave Reagent (Merck Millipore). Staining was analyzed using a Nikon TE 2000-E confocal microscope with 60× oil immersion objective lens (DIC).

### CytoTrack

One-hundred cancer cells were mixed with 500,000 PBMC. Cells were incubated with 250 nM rVAR2 for 30 min at 4 °C and secondarily with anti-penta His Alexa Fluor 488 (Cat. No. 35310, Qiagen, 1:500) and anti-human CD45 Cy5 (Cat. No. 19-0459, eBioscience, 1:10)^34^. After fixation in 4% formaldehyde, the cells were stained with DAPI (Cat. No. D1306, Life Technologies) and mounted on glass slides using FluorSave Reagent (Merck Millipore). rVAR2-positive cells were located using a CytoTrack CT4 Scanner. The resulting table of hotspots was subsequently analyzed for morphology and rVAR2, DAPI, and CD45 staining, as described^34^.

### Preparation of rVAR2- or anti-EpCAM antibody-coated beads

The recombinantly expressed VAR2CSA (rVAR2) protein used in the rVAR2 CTC isolation method was designed to include a 13-amino-acids peptide (SpyTag) from the fibronectin-binding protein Fba N-terminally, which enables covalent isopeptide bond formation to a biotinylated 12 kDa SpyCatcher protein^45^. The SpyCatcher was produced in *E. coli* Bl21 as a soluble poly-HIS tagged protein, and purified by Ni++ affinity chromatography. Purity was determined by SDS page and quality of protein was ensured by testing the capacity to form an isopeptide bond to a tagged protein. The tagged rVAR2 and biotinylated SpyCatcher fragment were incubated at room temperature for 1 h. After this step, the protein was incubated with CELLection™ Biotin Binder Dynabeads® (4.5 µm) at room temperature for at least 30 min resulting in rVAR2-coated beads (0.43 µg biotinylated protein per microliter bead suspension). For the EpCAM-based detection, CELLection™ Pan Mouse IgG Dynabeads® (4.5 µm) in combination with an anti-EpCAM antibody [Ber-EP4] (Cat. No. ab7504, abcam)^58^ were used. Anti-EpCAM antibody and beads were incubated for 30 min (0.02 µg anti-EpCAM antibody per microliter bead suspension). Remaining protein or antibody was removed by carefully washing the beads in PBS containing 0.1% BSA three times, each time using a neodymium magnet (10 × 12 mm) for dragging beads into a pellet.

### Cell culture and spike-in preparations

Prior to the spike-in experiments, PC3 cells or primary PDAC (PDX-derived) cells were harvested using trypsin−EDTA (Sigma-Aldrich) and resuspended in culture medium. Cell concentration was measured by manually counting the number of viable cells in a 1:1 mixture with Trypan Blue solution (Sigma-Aldrich). The suspensions were subsequently spiked into blood to achieve the desired concentrations.

GFP-expressing PDAC cells were used for the spike-in experiment with three and six cells. To ensure a precise cell count before spike-in, we did serial dilutions and placed the cells in a low-adhesion 96-well plate. After validation of the cell counts by microscopy, the cells were transferred to 5 mL of blood. These samples were then processed as described below with the exception of the CK staining.

### CTC isolation from blood

Blood samples were collected under the Barts Cancer Tissue Biobank Ethics committee protocol and informed written consent was obtained for all enrolled subjects. Blood was received in K2 EDTA tubes and processed within 2 h. The blood samples were divided into aliquots of 5 mL and red blood cells were lysed in 45 mL Red Blood Cell (RBC) lysis buffer containing 0.155 M ammonium chloride, 0.01 M potassium hydrogen carbonate, and 0.1 mM EDTA for 10 min. After centrifugation at 400×*g* for 8 min, the cell pellet was gently washed in PBS once. The centrifugation step was repeated, and finally cells were resuspended in RPMI medium containing 1% FBS in addition to 1 mM CaCl_2_ and 5 mM MgCl_2_ and transferred to a low retention microcentrifuge tube (Fisherbrand). Under these conditions, cells were incubated with ~1.6E6 rVAR2- or anti-EpCAM antibody-coated magnetic beads at 4 °C. Cancer cells adhering to beads were retrieved by running the isolation protocol on the IsoFlux™ machine (Fluxion). Isolated cancer cells were hereafter retrieved in 200 µL RPMI medium containing 1% FBS in addition to 1 mM CaCl_2_ and 5 mM MgCl_2_ and transferred to a low retention microcentrifuge tube (Fisherbrand). A neodymium cylinder magnet was used to drag cells bound to beads toward the bottom of the tube, enabling removal of the supernatant. The cells were then fixed in 4% PFA for 10 min and added onto glass slides, on which a circle with the same size as the magnet had been drawn using a water repellent Dako pen. When adding or removing buffer from the cells, the glass slide was placed on top of the magnet. The cells were blocked for 5 min in 10% normal donkey serum (NDS) prior to stain with PE-conjugated anti-CD45 [5B-1] antibody (Cat. No. 130-080-201, MACS Miltenyi Biotec, 1:100)^59^ for 30 min at room temperature. Hereafter, cells were permeabilized using 0.2% Triton X-100 diluted in PBS containing 0.5% BSA and 2 mM EDTA. This step was followed by staining of the cells with FITC-conjugated anti-CK [CK3-6H5] antibody (Cat. No. 130-080-101, MACS Miltenyi Biotec, 1:10)^60^ for 30 min at room temperature. To enable visualization of cell nuclei, the cells were stained with DAPI. The sample was mounted using Dako Faramount Aqueous Mounting Medium.

### Quantification of cancer cells

Enumeration of cancer cells was done manually using the Ariol image analysis system (Leica Biosystems Ltd., UK) with an Olympus BX61 microscope. Cancer cells were identified as CK+, CD45−, DAPI+ cells. PBMCs were identified as CK−, CD45+, DAPI+ cells.

### Four-color immunofluorescence staining on cancer cells

CTCs from prostate cancer patients isolated using rVAR2-coated beads were stained for EpCAM positivity using an anti-EpCAM antibody [Ber-EP4] (Cat. No. ab7504, abcam, 1:100) in combination with an Alexa Fluor 647-conjugated goat anti-Mouse IgG secondary antibody (Cat. No. A-21235, Invitrogen, 1:200). CTCs from prostate patients isolated using anti-EpCAM antibody-coated beads were stained for rVAR2 positivity using rVAR2 protein containing a V5-tag in combination with a FITC-conjugated anti-V5 antibody (Cat. No. R963-25, Invitrogen, 1:100)^26^. For these samples, the CK positivity was observed using an anti-CK (CK3-6H5) antibody (Cat. No. 130-090-866, MACS Miltenyi Biotec, 1:10) in combination with an Alexa Fluor 647-conjugated goat anti-Mouse IgG secondary antibody (Cat. No. A-21235, Invitrogen, 1:200)^61^.

For vimentin staining, cells were stained with FITC-conjugated anti-CK (CK3-6H5) antibody (Cat. No. 130-080-101, MACS Miltenyi Biotec, 1:10), and anti-vimentin (EP21) antibody (Cat. No. AC0024, Epitomics, 1:50) in combination with an Alexa Fluor 647-conjugated anti-rabbit secondary antibody (Cat. No. A-31573, Invitrogen, 1:200).

### Verification of PDAC CTCs using ddPCR

Total genomic DNA (gDNA) was extracted directly from the sample material on the slide used for enumeration (QIAamp DNA Micro Kit, Qiagen). The QX100 Droplet Digital PCR System (ddPCR, Biorad), PrimePCR *KRAS* mutant, and WT assays (Biorad, dHsaCP2000001 (G12D), dHsaCP2000002 (G12D WT), dHsaCP2000005 (G12V), dHsaCP2000006 (G12V WT), dHsaCP2000009 (G12R), and dHsaCP2000010 (G12R WT)) were used to detect the following *KRAS* mutations in gDNA: G12D, G12V, and G12R. A total 50 ng of gDNA was used for each PCR. PDAC 215 (G12D), PDAC 247 (G12V), and PDAC JH033 (G12R) were used for positive controls and leukocytes of a healthy donor served as a negative control. In ddPCR, the samples containing gDNA were partitioned into 20,000 droplets and loaded into thermo cycler. Following PCR amplification, droplets from each sample are streamed in single file through the droplet reader. Absolute concentration of *KRAS* mutant and WT DNA copies were determined using the QuantaSoft software provided by the manufacturer. Briefly, positive droplets which contain at least one copy of the target exhibit increased fluorescence. The system detects Mutant (HEX) and WT (FAM) alleles by counting the number of droplets positive for each fluorophore.

### Single cell isolation using the CellCelector

CTCs were immunofluorescently stained with FITC-conjugated anti-CK [CK3-6H5] antibody (Cat. No. 130-080-101, MACS Miltenyi Biotec, 1:10), PE-conjugated anti-CD45 [5B-1] antibody (Cat. No. 130-080-201, MACS Miltenyi Biotec, 1:100), EpCAM [Ber-EP4] (Cat. No. ab7504, Abcam, 1:100) with Goat anti-Mouse Alexa Fluor® 647 (AF647) secondary antibody (Cat. No. A-21235, ThermoFisher, 1:200), and DAPI.

Single cells were isolated using an automated micromanipulator, CellCelector (ALS GmbH, Jena, Germany). This system consists of an inverted fluorescent microscope (CKX41, Olympus, Tokyo, Japan) with a CCD camera system (XM10-IR, Olympus, Tokyo, Japan) and a vertical 30 µm glass capillary on a robotic arm. ALS CellCelector Software 3.0 (ALS, Jena, Germany) was used for analysis. Labeled cell solutions were transferred to a glass slide and cells were allowed to settle. The cells were visualized by bright field (BF) and fluorescent microscopy for nuclear DAPI and CD45-PE staining to verify morphology and CD45 negativity at 20× magnification. Then CK+EpCAM+ and CK+EpCAM− target cells were detected in the FITC and AF647 channel at 40× magnification. The target cells were detected by the software following pre-defined settings, manually approved, and then fully automatically aspirated and transferred into PCR tubes containing 100 μl of lysis buffer of the Guanidine Thiocynate (GTC) Method. Total RNA was isolated by the GTC method using standard protocols^62^. The purified RNA was used for cDNA synthesis using the SuperScript VILO Master Mix according to the manufacturer’s recommendations (Cat. No. 1455280, Invitrogen), followed by preamplification using AmpliTaq Gold 360 Master Mix (Cat. No. 4398881, Applied Biosystems), and 200 nM each of forward and reverse primers for *KRAS* [5′-CTGAAAATGACTGAATATAAACTTGTGG-3′ (forward) and 5′-TAGCTGTATCGTCAAGGCACTC-3′ (reverse)]. Preamplified DNA was used for *KRAS* mutation detection by ddPCR. Consistent with the analysis of total genomic CTC extract, the PrimePCR *KRAS* mutant and WT assays (Biorad, dHsaCP2000001 (G12D), dHsaCP2000002 (G12D WT), dHsaCP2000005 (G12V), and dHsaCP2000006 (G12V WT)) were used to detect the G12D and G12V *KRAS* mutations.

### CellSearch

The identification of CTCs utilizing the CellSearch® CTC platform (Celltracks Autoprep and Celltracks Analyzer II) was performed as per the manufacturer’s instructions. Informed consent was obtained from all subjects and blood samples were collected in CellSave tubes. The CTC assay was performed with the CellSearch® CTC Kit, which contains reagents for immunomagnetic isolation of EpCAM-positive cells. The isolated cells were stained with DAPI, PE-conjugated CK monoclonal antibodies, and APC-labeled CD45 monoclonal antibody (CELLTRACKS® AUTOPREP® System).

### Statistics

STATA 14 software was used for all analyses. The effect on rVAR2 binding by treating A549 cells with TGFβ was tested in three independent experiments by declaring MFI data to be panel data and testing the effect of TGFβ treatment versus control in a generalized least squares regression model using the xtgls command including treatment group and ln(rVAR2) concentration as explanatory variables (Fig. 2c).

The ability of the anti-EpCAM and rVAR2 assays to capture CTC from patient samples were compared by Wilcoxon rank sum test for paired data. *P* values are from two-sided tests (Fig. 4d–f). The association between cancer stage and number of CTC detected by rVAR2 was tested by Kruskal–Wallis test followed by comparing groups by the Mann–Whitney *U* test (Fig. 5b).

### Data availability

Data supporting the findings of this study are within this manuscript or available from the corresponding authors upon reasonable request.

## Electronic supplementary material

Supplementary Information
