# Author Correction: The VAR2CSA malaria protein efficiently retrieves circulating tumor cells in an EpCAM-independent manner

**DOI:** 10.1038/s41467-022-29351-z

**Published:** 2022-06-07

**Authors:** Mette Ø. Agerbæk, Sara R. Bang-Christensen, Ming-Hsin Yang, Thomas M. Clausen, Marina A. Pereira, Shreya Sharma, Sisse B. Ditlev, Morten A. Nielsen, Swati Choudhary, Tobias Gustavsson, Poul H. Sorensen, Tim Meyer, David Propper, Jonathan Shamash, Thor G. Theander, Alexandra Aicher, Mads Daugaard, Christopher Heeschen, Ali Salanti

**Affiliations:** 1 Centre for Medical Parasitology at Department of Immunology and Microbiology, University of Copenhagen and Department of Infectious Diseases, Copenhagen University Hospital, 2200 Copenhagen, Denmark; 2 Vancouver Prostate Centre, Vancouver, BC V6H 3Z6 Canada; 3 Department of Urologic Sciences, University of British Columbia, Vancouver, BC V5Z 1M9 Canada; 4 Stem Cells in Cancer & Ageing, Barts Cancer Institute, Queen Mary University of London, London, EC1M 6BQ United Kingdom; 5 Division of Urology, Department of Surgery, Tri-Service General Hospital, National Defense Medical Center, 11490 Taipei, Taiwan; 6 Department of Molecular Oncology, British Columbia Cancer Research Centre, Vancouver, BC V5Z 1L3 Canada; 7 UCL Cancer Institute, University College London, London, WC1E 6BT United Kingdom; 8 Department of Medical Oncology, Barts Health NHS, London, EC1A 7BE United Kingdom; 9 School of Medical Sciences, University of New South Wales, Sydney, NSW 2052 Australia

Correction to: *Nature Communications* 10.1038/s41467-018-05793-2, published online 16 August 2018.

This Article contained an error in the consent of some of the patients used in Figure 4. Following an institute-led investigation within BARTS Cancer Institute post-publication, no documentation of informed consent from the nine lung cancer patients whose blood samples were used in this research project could be recovered and therefore, this data have been removed from the published article. The patients and their families were informed of the original error and apologies were made.

The following changes have been made to the paper to remove all mention of the lung cancer samples and the data associated with them.

In the abstract, the sentence ‘We show that rVAR2 efficiently captures CTCs from hepatic, lung, pancreatic, and prostate carcinoma patients with minimal contamination of peripheral blood mononuclear cells.’ has been changed to read ‘We show that rVAR2 efficiently captures CTCs from hepatic, pancreatic, and prostate carcinoma patients with minimal contamination of peripheral blood mononuclear cells.’

A revised Figure 4 excluding the lung cancer patients is shown below, this includes a revised version of Fig. 4a without the lung cancer samples and the removal of Fig. 4c, 4f, and 4i.
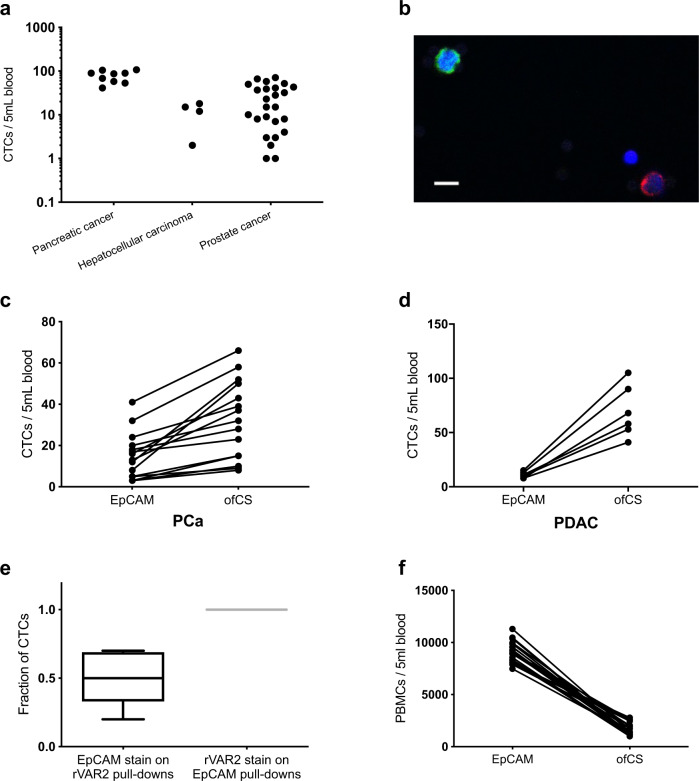


In the results sections ‘rVAR2-coated beads capture CTCs in patient blood samples’ the following sentence ‘In order to test whether rVAR2-coated beads enabled the isolation of CTCs from clinical samples, we analyzed blood samples from patients with pancreatic (*n* = 9), hepatic (*n* = 4), prostate (*n* = 25), and lung (*n* = 6) cancer at different stages of disease. rVAR2-coated beads captured CK+, CD45, DAPI+ cells in all four cancer types (Fig. 4a–c), whereas no CTCs were detected in blood samples from healthy donors (*n* = 16)’ has been amended to read ‘In order to test whether rVAR2-coated beads enabled the isolation of CTCs from clinical samples, we analyzed blood samples from patients with pancreatic (*n* = 9), hepatic (*n* = 4), and prostate (*n* = 25) cancer at different stages of disease. rVAR2-coated beads captured CK+, CD45, DAPI+ cells in all three cancer types (Fig. 4a–b), whereas no CTCs were detected in blood samples from healthy donors (*n* = 16).

In the results section ‘rVAR2 captures more CCs and less PBMCs’ the sentence ‘To directly compare our ofCS-targeting CTC isolation method with the more common EpCAM-targeting strategy, we aimed to capture CTCs in a subset of the blood samples from lung, prostate, and pancreatic cancer patients using either rVAR2-coated or anti- EpCAM antibody-coated beads on the IsoFlux™ system. ‘has been amended to read ‘To directly compare our ofCS-targeting CTC isolation method with the more common EpCAM-targeting strategy, we aimed to capture CTCs in a subset of the blood samples from prostate, and pancreatic cancer patients using either rVAR2-coated or anti-EpCAM antibody-coated beads on the IsoFlux™ system. The sentence **‘**The rVAR2-based method detected higher CTC numbers than the EpCAM-based method in all patient-matched blood samples (Fig. 4d-f)’ has been amended to read ‘The rVAR2-based method detected higher CTC numbers than the EpCAM-based method in all patient-matched blood samples (Fig. 4c-d).’

The sentence ‘On average the rVAR2-based CTC isolation resulted in a 5.3×, 2.8×, or 6.4× higher CTC levels for lung (*n* = 4), prostate (*n* = 15), and pancreatic (*n* = 6) cancer, respectively.’ Has been amended to read ‘On average the rVAR2-based CTC isolation resulted in a 2.8×, or 6.4× higher CTC levels for prostate (*n* = 15), and pancreatic (*n* = 6) cancer, respectively.

‘The sentence ‘Only half of the rVAR2-captured CTCs were EpCAM positive, whereas all cells retrieved by the EpCAM-based method were ofCS positive as determined by rVAR2 staining (Fig. 4g). has been amended to read ‘Only half of the rVAR2-captured CTCs were EpCAM positive, whereas all cells retrieved by the EpCAM-based method were ofCS positive as determined by rVAR2 staining (Fig. 4e). The sentence ‘The higher MAF of the rVAR2-based CTC isolations most likely reflects the higher number of isolated CTCs as well as a lower PBMC contamination (Fig. 4h).’ has been amended to read The higher MAF of the rVAR2-based CTC isolations most likely reflects the higher number of isolated CTCs as well as a lower PBMC contamination (Fig. 4f).’

In the results section “rVAR2 can be used for CTC isolation in early-stage cancer”, the sentences ‘Nevertheless, the EpCAM-based capture analyzed on the IsoFlux™ detected 5, 8, 5, and 5 CTCs in the patient-matched samples (estimated from the 5 mL capture as shown in Fig. 4d). Interestingly, the rVAR2-based detection method captured the highest number of cells with 12, 23, 15, and 23 CTCs, respectively, in the four patient samples (estimated from the 5 mL capture as shown in Fig. 4d) have been amended to read ‘Nevertheless, the EpCAM-based capture analyzed on the IsoFlux™ detected 5, 8, 5, and 5 CTCs in the patient-matched samples (estimated from the 5 mL capture as shown in Fig. 4c). Interestingly, the rVAR2-based detection method captured the highest number of cells with 12, 23, 15, and 23 CTCs, respectively, in the four patient samples (estimated from the 5 mL capture as shown in Fig. 4c).

The following paragraph, ‘As our results indicated that rVAR2-captured CTCs could contain a mesenchymal subpopulation, we stained CTC enriched samples from two lung cancer patients for the mesenchymal marker vimentin. Samples from both patients contained cells that were double positive for CK and vimentin, strongly supporting our hypothesis that rVAR2 efficiently captures CTCs with an intermediate epithelial and mesenchymal phenotype (Fig. 4i). Notably, a minor fraction of the double-negative cells (CK+, CD45+, DAPI+) was found to be vimentin positive, suggesting the presence of mesenchymal CK+ CTCs within the rVAR2-enriched cell population.’ which was the final paragraph of the results section entitled ‘rVAR2 captures more CTCs and less PBMCs’, has been removed from the Article.

The following sentences in the Discussion section ‘Importantly, this protocol enabled isolation of CTCs from pancreatic, hepatic, lung, and prostate cancer patients at various stages of disease, illustrating the broad applicability of the rVAR2-based CTC isolation method. Furthermore, we showed that rVAR2 resulted in a higher CTC yield in blood samples from prostate, pancreatic, and lung cancer patients as compared to EpCAM-based isolation of patient-matched samples.’ have been amended to read ‘Importantly, this protocol enabled isolation of CTCs from pancreatic, hepatic and prostate cancer patients at various stages of disease, illustrating the broad applicability of the rVAR2-based CTC isolation method. Furthermore, we showed that rVAR2 resulted in a higher CTC yield in blood samples from prostate and pancreatic cancer patients as compared to EpCAM-based isolation of patient-matched samples.’

The following sentence ‘In line with this, we demonstrate that the rVAR2-enriched CTC population from cancer patients contains vimentin-positive cells as well as EpCAM-negative cells, indicating that the rVAR2 isolates include mesenchymal-like subpopulations.’ which was after the sentence ‘In contrast, we show that rVAR2 binding to cancer cells is maintained after induction of EMT.’ has been removed from the Discussion section.

The following sentences ‘Intriguingly, Vim+, CK_, CD45_ cells were detected in two blood samples from non-small cell lung cancer (NSCLC) patients and they may represent mesenchymal CTCs. Future studies will have to define and further validate the nature of the captured vimentin+, CK− putative CTCs.’ which were after the sentence ‘Thus, it is likely that capturing CTCs with rVAR2 followed by CK detection will miss certain subsets of CTCs.’ have been removed from the Discussion section.

In the methods section, the following paragraph, ‘For vimentin staining, cells were stained with FITC-conjugated anti-CK (CK3-6H5) antibody (Cat. No. 130-080-101, MACS Miltenyi Biotec, 1:10), and antivimentin (EP21) antibody (Cat. No. AC0024, Epitomics, 1:50) in combination with an Alexa Fluor 647-conjugated anti-rabbit secondary antibody (Cat. No. A-31573, Invitrogen, 1:200). which was at the end of the methods section entitled ‘Four-color immunofluorescence staining on cancer cells’ has been removed

The sentence in the ‘Statistics’ section of the Methods ‘The ability of the anti-EpCAM and rVAR2 assays to capture CTC from patient samples were compared by Wilcoxon rank sum test for paired data. P values are from two-sided tests (Fig. 4d–f). ‘has been amended to ‘The ability of the anti-EpCAM and rVAR2 assays to capture CTC from patient samples were compared by Wilcoxon rank sum test for paired data. P values are from two-sided tests (Fig. 4c and d).

The Figure 4 legend ‘rVAR2- and EpCAM-based CTC isolation and enumeration in cancer patients. a Number of CTCs isolated from 5 mL pancreatic (*n* = 9), hepatocellular (*n* = 4), prostate (*n* = 25), and lung (*n* = 6) cancer patient-derived blood using rVAR2-coated beads. CTCs were enumerated by immunofluorescence stainings and defined as CK+ CD45_ DAPI+. b Representative confocal microscopy image of a circulating tumor cell isolated with rVAR2 from blood derived from one of the pancreatic cancer patients (patient 4, Table 3). Isolated cells were stained with anti-cytokeratin FITC antibody (green), anti-CD45 PE antibody (red), and DAPI (blue). Scale bar, 10 µm. c Representative confocal microscopy image of circulating tumor cells isolated with rVAR2 from blood derived from one of the non-small cell lung cancer (NSCLC) patients. Isolated cells were stained with anti-cytokeratin FITC antibody (green), anti-CD45 PE antibody (red), and DAPI (blue). Scale bar, 10 µm. d Number of CK+ CD45_ DAPI+ CTCs isolated using rVAR2 or anti-EpCAM antibody-coated beads from 5 mL blood from 15 of the stage II_III prostate cancer (PCa) patients (*P* < 0.02, Wilcoxon test for paired data). e Number of CK+ CD45_ DAPI+ CTCs isolated using rVAR2 or anti-EpCAM antibody-coated beads from 5 mL blood from six of the stage III–IV pancreatic ductal adenocarcinoma (PDAC) patients. f Number of CK+ CD45_ DAPI+ CTCs isolated using rVAR2 or anti-EpCAM antibody-coated beads from 5 mL blood from four of the stage IV NSCLC patients. g Box-Whiskers plot showing post-isolation characterization of CK+ CD45_ DAPI+ CTCs using EpCAM or rVAR2 stain on CTCs isolated using rVAR2 (*n* = 7) or anti-EpCAM antibody-coated (*n* = 7) beads, respectively. The median is presented as the center line, whiskers as min to max values, and the 25th to 75th percentiles define the box. h Number of PBMCs contaminating the isolated CTCs from patient-matched blood samples using rVAR2 or anti-EpCAM antibody-coated beads. PBMC levels were estimated by imunofluorescence stainings and defined as CK_, CD45+, DAPI+ stained cells (*P* < 0.0001, Wilcoxon test for paired data) (*n* = 23). i Number of anti-CK and anti-vimentin antibody stained cells in the rVAR2-based CTC enrichments from 5 mL blood samples from two of the patients with stage IV NSCLC ’ has been amended to read ‘Fig. 4 rVAR2- and EpCAM-based CTC isolation and enumeration in cancer patients. a Number of CTCs isolated from 5 mL pancreatic (*n* = 9), hepatocellular (*n* = 4), and prostate (*n* = 25) cancer patient-derived blood using rVAR2-coated beads. CTCs were enumerated by immunofluorescence stainings and defined as CK+ CD45− DAPI+. b Representative confocal microscopy image of a circulating tumor cell isolated with rVAR2 from blood derived from one of the pancreatic cancer patients (patient 4, Table 3). Isolated cells were stained with anti-cytokeratin FITC antibody (green), anti-CD45 PE antibody (red), and DAPI (blue). Scale bar, 10 μm. c Number of CK+ CD45− DAPI+ CTCs isolated using rVAR2 or anti-EpCAM antibody-coated beads from 5mL blood from 15 of the stage II−III prostate cancer (PCa) patients (*P* < 0.02, Wilcoxon test for paired data). d Number of CK+ CD45− DAPI+ CTCs isolated using rVAR2 or anti-EpCAM antibody-coated beads from 5mL blood from six of the stage III–IV pancreatic ductal adenocarcinoma (PDAC) patients. e Box-Whiskers plot showing post-isolation characterization of CK+ CD45− DAPI+ CTCs using EpCAM or rVAR2 stain on CTCs isolated using rVAR2 (*n* = 7) or anti-EpCAM antibody-coated (*n* = 7) beads, respectively. The median is presented as the center line, whiskers as min to max values, and the 25th to 75th percentiles define the box. f Number of PBMCs contaminating the isolated CTCs from patient-matched blood samples using rVAR2 or anti-EpCAM antibody-coated beads. PBMC levels were estimated by immunofluorescence stainings and defined as CK−, CD45+, DAPI+ stained cells (*P* < 0.0001, Wilcoxon test for paired data) (*n* = 23).’

Removing the lung cancer samples does not affect the overall conclusions of the study. The PDF and HTML versions of the Article have been updated.

