# Supplementary material for: The VAR2CSA malaria protein efficiently retrieves circulating tumor cells in an EpCAM-independent manner
Source: Nat Commun. 2018 Aug 16;9:3279. doi: 10.1038/s41467-018-05793-2 (PMC6095877; doi:10.1038/s41467-018-05793-2)
Supplement: Supplementary file 1 — Supplementary Information [file 41467_2018_5793_MOESM1_ESM.pdf]

Supplementary information

The VAR2CSA malaria protein efficiently retrieves circulating tumor cells in an EpCAM-independent manner

Agerbæk, Bang-Christensen, Yang, Clausen et al.

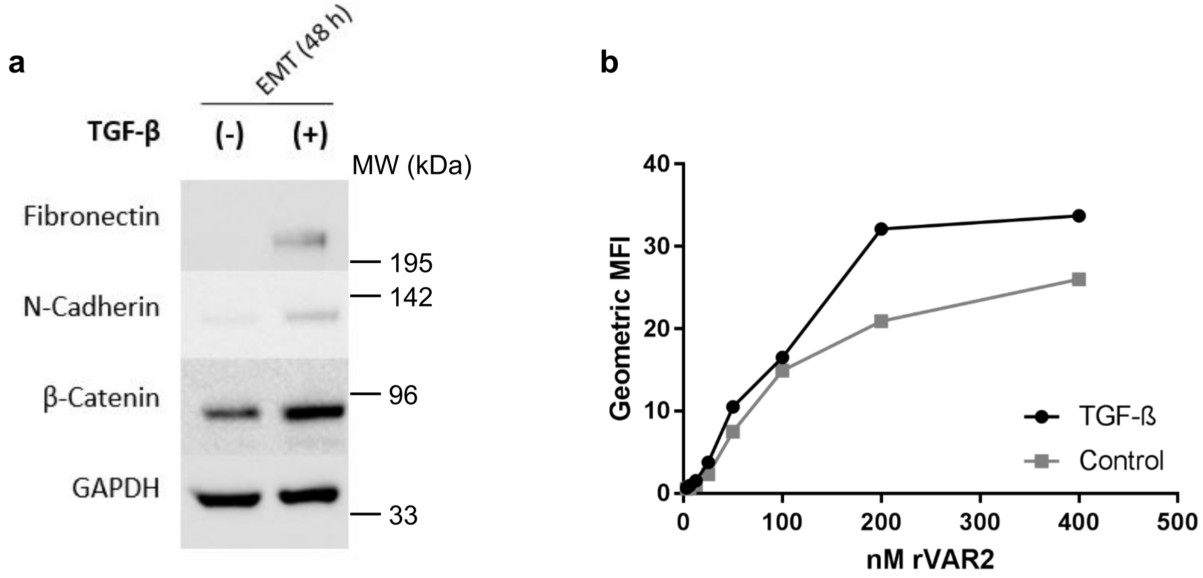

Supplementary Figure 1

In vitro EMT assay.

a Western blot of U87mg cell lysates after 48 hours of treatment with TGFβ (EMT(+)) or control (TGFβ dissolution buffer) (EMT(-)). The blot was incubated with anti-Fibronectin (240 kDa), anti-N-Cadherin (140 kDa), anti-β-Catenin (92 kDa), or anti-GAPDH (37 kDa) antibodies and detected by anti-rabbit HRP antibody or anti-mouse HRP antibody in the case of Fibronectin. Detection of GAPDH served as a loading control.

b Geometric mean fluorescence intensity (MFI) of rVAR2 binding to U87mg cells treated with TGFβ or control (TGFβ dissolution buffer) for 48 hours. Binding was measured by flow cytometry.

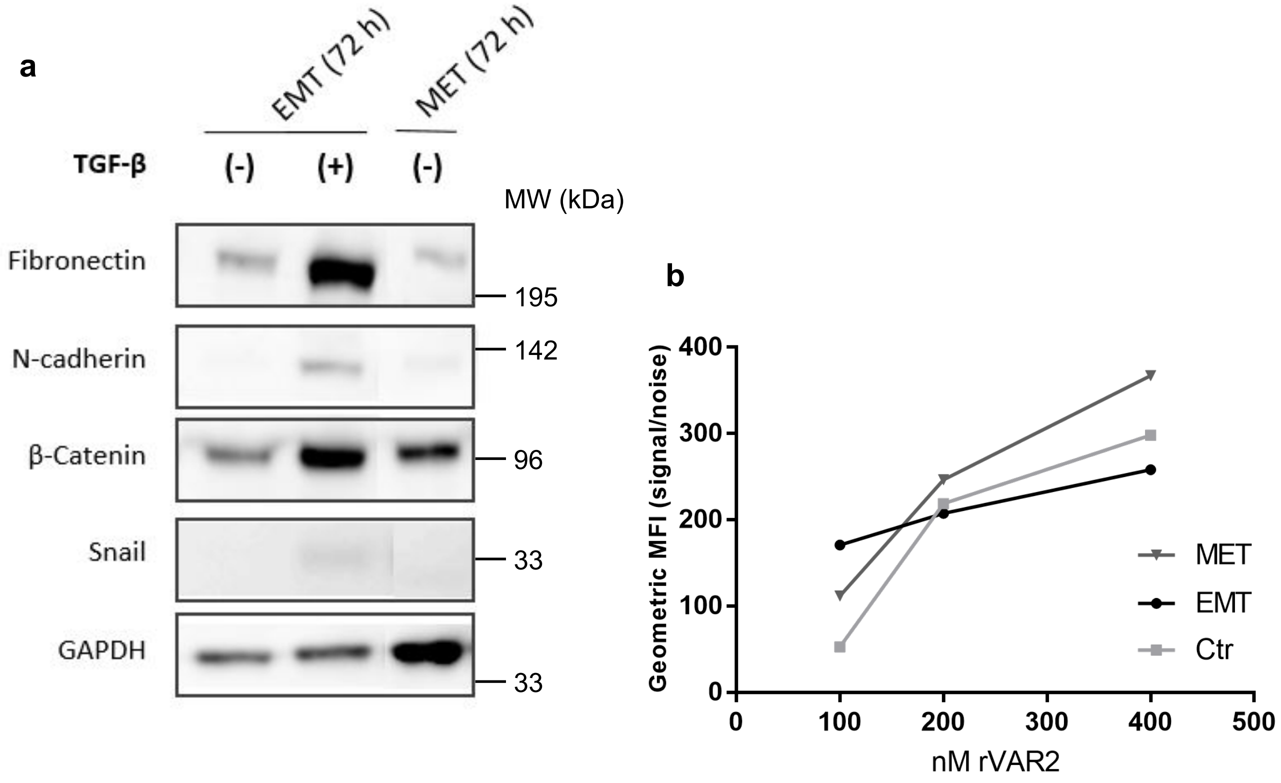

Supplementary Figure 2

In vitro EMT-MET assay.

a Western blot of A549 cell lysates after 72 hours of treatment with TGFβ (EMT (+)) or control (TGFβ dissolution buffer) (EMT (-)). After EMT induction, TGFβ was removed and the cells were left under normal culture conditions for another 72 hours (MET (-)). The blot was incubated with anti-Fibronectin (240 kDa), anti-N-cadherin (140 kDa), anti-β-catenin (92 kDa), anti-Snail (29 kDa) or anti-GAPDH (37 kDa) antibodies and detected by anti-rabbit HRP antibody or anti-mouse HRP antibody in the case of Fibronectin. Detection of GAPDH served as a loading control.

b Geometric mean fluorescence intensity (MFI) (signal/noise) of rVAR2 staining of A549 cells treated with TGFβ or control (TGFβ dissolution buffer) for 72 hours (EMT and Ctr, respectively) and of A549 cells after return to their epithelial state, by removal of the EMT-inducer TGFβ (MET). Binding was measured by flow cytometry.

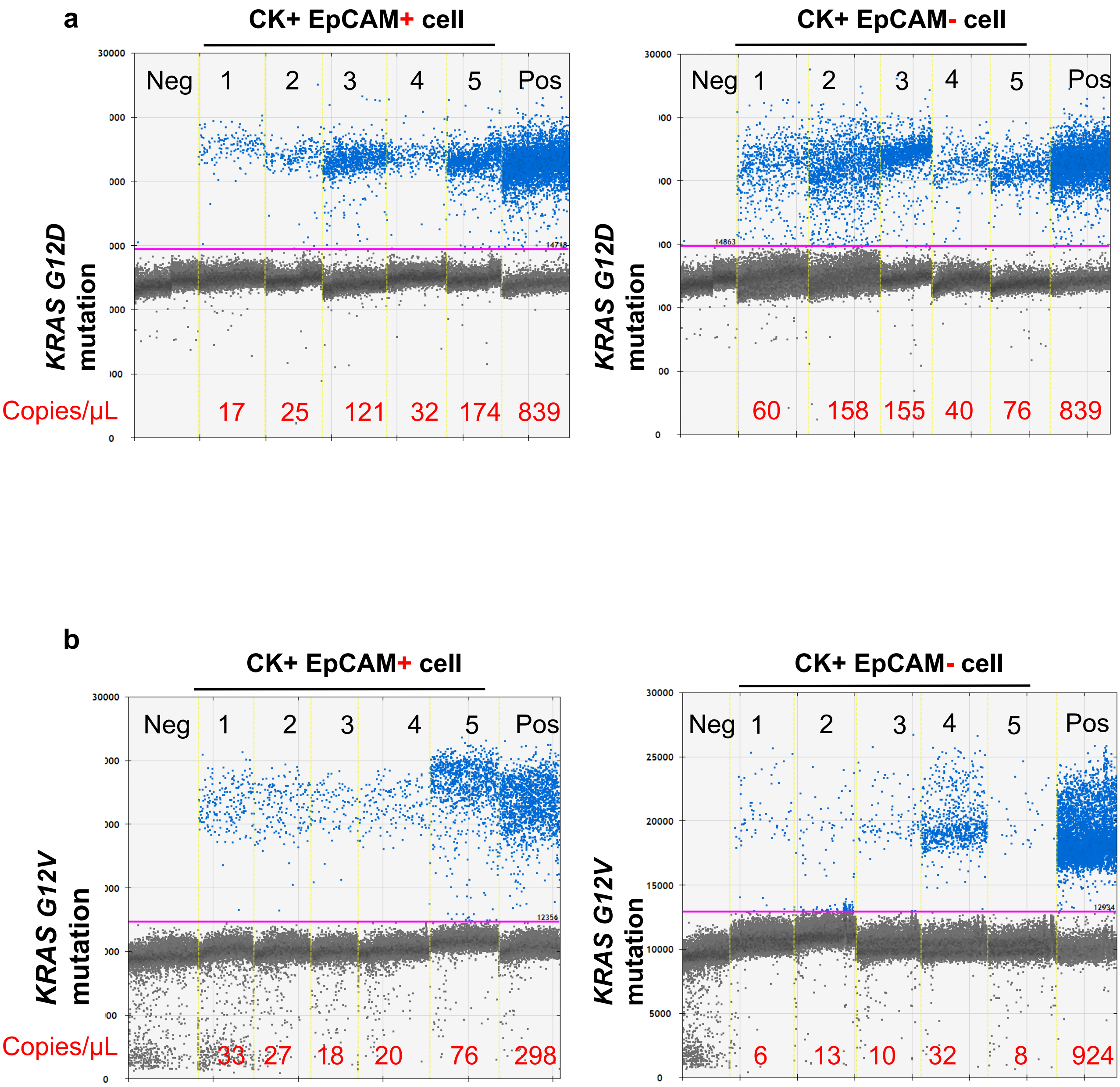

Supplementary Figure 3

Single cell KRAS mutation analysis of CTCs from two PDAC blood samples.

a *KRAS G12D* mutations measured by digital droplet PCR (ddPCR) for pure single CK+EpCAM+ vs. CK+EpCAM- CTCs (1-5) from patient 1 sorted by CellCelector system. Total RNA was isolated and was used for cDNA synthesis, followed by preamplification prior to the ddPCR run. Numbers of DNA copies per microliter are shown as insert. DNA extracts from PBMCs and PDAC cells were used as negative and positive controls, respectively.

b *KRAS G12V* mutations measured for 5 EpCAM positive and 5 EpCAM negative cells from patient 2 as in (a).

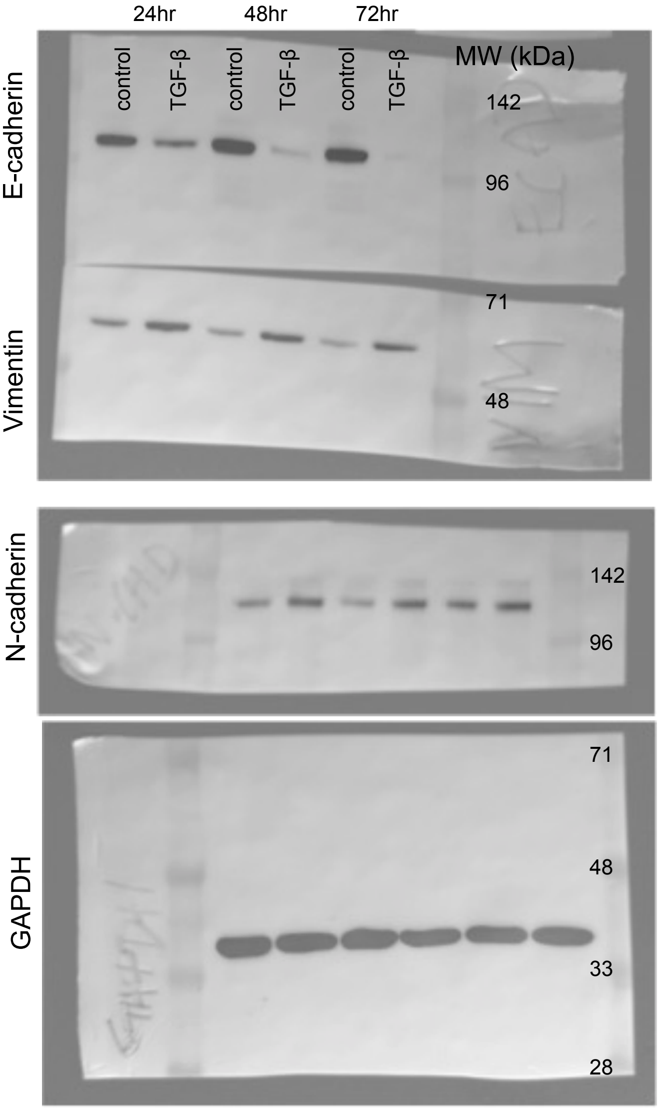

Supplementary Figure 4

Epithelial-mesenchymal transition increases rVAR2 binding.

a Uncropped scans of western blots presented in figure 2a. Western blot of A549 cell lysates after 24, 48 and 72 hours of treatment with TGF-β or control (TGF-β dissolution buffer). Blots were incubated with rabbit anti-E-cadherin, anti-Vimentin, anti-N-cadherin or anti-GAPDH antibodies and detected by anti-rabbit HRP antibody.
